# Why should I switch on my camera? Developing the cognitive skills of compassionate communications for online group/teamwork management

**DOI:** 10.3389/fpsyg.2023.1113098

**Published:** 2023-08-04

**Authors:** J. M. P. V. K. Jayasundara, Theo Gilbert, Saskia Kersten, Li Meng

**Affiliations:** ^1^Department of English Language and Linguistics, School of Creative Arts, University of Hertfordshire, Hatfield, United Kingdom; ^2^Department of English Language Teaching, Uva Wellassa University, Badulla, Sri Lanka; ^3^The Learning and Teaching Innovation Centre, University of Hertfordshire, Hatfield, United Kingdom; ^4^Department III—English and American Studies, Institute of English Philology, Ludwig-Maximilians-Universität, Munich, Germany; ^5^School of Physics, Engineering and Computer Science, University of Hertfordshire, Hatfield, United Kingdom

**Keywords:** compassionate communication, team/group work, online, social experience, learning experience

## Abstract

**Introduction:**

Associated with learning and social isolation from each other during the pandemic-driven transition to online platforms in Higher Education (HE), many students were, and remain, reluctant to turn on their video cameras to be present with each other during their online meetings.

Using the Compassionate Mind Foundation's definition of compassion, not as an emotion, but as a psychobiological motivation to take wise action to help when self or others struggle, this comparative study examined (a) the deployment by students during online, task-focused group/team meetings, of taught verbal and non-verbal communication strategies that were explicitly compassionate and (b) the effects of these strategies on each other's social and learning experiences in these meetings, compared to when they did not use them.

**Methods:**

Twenty-four STEM students from a sample of five Sri Lankan universities, were mixed, then divided into six groups of four students per group. This mixed-methods study, video-recorded and analyzed each group's task-focused group meetings before, then after, an online interactive 90-min training session (the intervention) in the Cognitive Skills of Compassionate Communications (CSCC) for groups/teams.

**Results:**

Using R, SPSS and Microsoft Excel to analyse the quantitative data, a statistically significant improvement in students' screen-gaze attentiveness was identified after the CSCC intervention. The qualitative data analysis explained this and other behavioral changes that were shown to enhance students' social and learning experiences in their online meetings.

Given the strong historical and political drivers of current divisions across Sri Lankan student communities, these findings call for more urgent research on compassion as a cognitive competence for accelerating group/team cohesion and criticality across HE, and beyond.

## 1. Introduction

The COVID-19 pandemic has substantially affected the education sector due to the sudden unexpected pandemic-driven shift to online teaching and learning irrespective of the readiness of many teachers and learners. Hence, students' disconnection (Bauer et al., 2020; Stanford University, 2020; Schwenck and Pryor, 2021) has impeded teaching and learning effectiveness and student social connectedness with each other (Lin et al., 2021). The impact on students' confidence, the lowering of their overall cognitive performances, and associated costs to students' quality of life were highlighted as major consequences of this (Aleman and Sommer, 2020). Recent research highlights some of the causes of students' reluctance to turn on their cameras, such as shyness, privacy concerns, peer pressure to talk when the camera is on, and self-perceptions of less-than-optimal personal appearance (Zhao et al., 2020; Lin et al., 2021). In addition to these factors, students also express concerns regarding the intrusion into their home environments (Gherheş et al., 2021).

This study argues that students' expressed reluctance to turn on their cameras for the above-mentioned reasons and thus being observed by others when participating in online teaching and learning settings worsened their isolation during the pandemic (Castelli and Sarvary, 2021). Relatedly, the non-use of cameras negatively affected both teacher–student and student–student interaction because observation of students' non-verbal communications during online meetings was not always possible, which in turn likely weakened the quality of their verbal communications in relation to their social and learning aspects (Zhao et al., 2020). This is at least in part because not being able to see participants led to teachers not being able to check students' understanding by paying attention to their body language, especially in terms of student facial expressions, and Palacios et al. (2022) note students' difficulties to perform as a group when some members kept their cameras off.

Several possible solutions have been suggested to encourage students to engage in active learning in online communities (Katchen, 1992; Cacioppo and Hawkley, 2009; Hawkley and Cacioppo, 2010; Leung et al., 2021; Schwenck and Pryor, 2021), including encouraging the use of microphones, asking questions unrelated to the target subject to break the ice and make them feel comfortable (Palacios et al., 2022), and making it mandatory for students to switch on their cameras during online classes to motivate them to stay focused (Lin et al., 2021).

Yet, in all this, there is very little discussion of the explicit role of compassion, which is empirically defined as a cognitive, psychobiological motivation (Gilbert, 2019), and its role in enhancing self and others' learning and social connectedness in online group meetings.

Gilbert (2016, 2017) and Harvey et al. (2020) investigated the learning and social cohesion among student team members during in-person classes after receiving compassionate communications training and found that learning and social cohesion were enhanced by it. In that training, as in the present study here, students were taught practical strategies to dismantle the two behaviors that they had ranked the most problematic in teamwork meetings, which were a tendency by some team members to either (a) over talk, or “monopolise” the group (Yalom and Leszsz, 2005) so that others had little chance to speak, or (b) say little or nothing, thus contributing very little to the group. Similarly, Jayasundara et al. (2022) demonstrated the feasibility and value of developing Cognitive Skills of Compassionate Communications (CSCC) among UK HE STEM students in their online group work management. This latter study (Jayasundara et al., 2022) identifies how students were motivated to turn on their webcams in their online group meetings after recognizing their own abilities to support one another through both their verbal and non-verbal compassionate communications during their team meetings.

The current study was conducted to investigate whether, and, if so, how, the above understanding of compassion is relevant to Sri Lankan-based HE STEM students in Sri Lankan universities despite clear evidence of tensions between Sinhalese, Tamil, and Muslim students in the country.

Hence, the aspect of the study presented here investigates the adaptability of evidence-based CSCC strategies among Sri Lankan HE STEM students considering them as a suitably challenging choice to explore the applicability of CSCC for enhancing their group cohesion along with learning experience. This is because of the well-documented tensions among Sinhalese, Tamil, and Muslim students in Sri Lanka. British Empire imperialism and its divisive legacy culminated in the country's 26-year civil war (1983–2009; Gunasingam, 1999; Subramanian, 2015). Even after this, intersectional violence throughout the nation—including among Sri Lankan HE students—remains an issue. As recently as 2019 (AdaDerana, 2019a,b; Alwis, 2019), the Sri Lankan government was forced to close all 15 state universities due to the Easter bombings across the country by Muslim extremists. Some state universities were closed for as long as 2 months, only for some of these state universities to be closed yet again when student conflicts re-ignited after their return to campuses. This wider socio-economic political and historical context has been an obvious activator of the collective threat and drive systems, that are explained next, across whole communities in Sri Lanka.

### 1.1. The theoretical model

**Figure 1:**
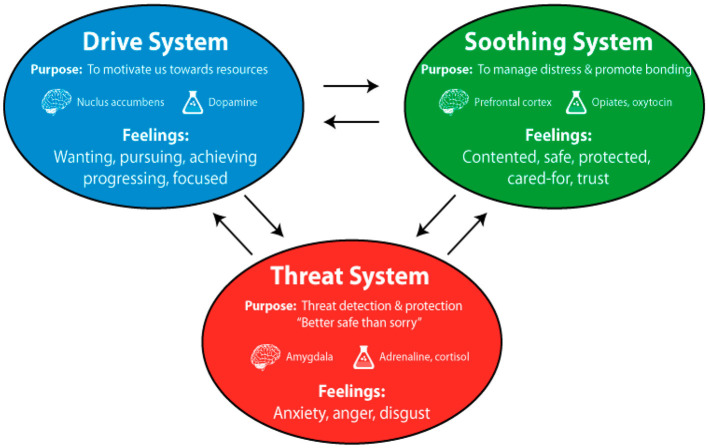
The brain's emotion regulation system. Source: adapted by Mindfulness and Clinical Psychology Solutions (2019) from Gilbert (2005, p. 3).

The psychobiological model of compassion (Compassionate Mind Foundation) was used as the theoretical model to map the wider socio, economic, political, and historical context in Sri Lanka to the current study. As the Compassionate Mind Foundation explains, humans switch between three mood-regulating systems: threat, drive, and soothing (Figure 1).

Each of these three systems is associated with different brain regions and different brain chemistry. Systemic imbalance among the three leads to distress, which is frequently correlated with the underdevelopment of the soothing system (Compassionate Mind Foundation; Mindfulness Clinical Psychology Solutions, 2019). Our brains are hard-wired to be alert to threats (fight, flight, and freeze). This threat system has enabled countless species to survive. The trouble is that in modern humans, this system can disable the brain's capacity to engage in higher-order thinking such as decision-making and problem-solving (Cozolino, 2013). The second mood-regulatory system—the drive system—enables us to strive to achieve what we want or need (or what we believe we need). But overstimulation of the threat and drive systems, e.g., in group monopoliser, leads to an imbalance between the three mood regulatory systems; the soothing system is underdeveloped. This may cause distress or psychological difficulties to both groups and individuals. The soothing system, the third mood-regulatory system in the brain, is activated by giving or receiving care from self or others and allows us to think more calmly, more rationally, and focus. The soothing system of the brain can be trained to facilitate a balance among these three systems (Gilbert et al., 2009).

The balance among these three systems becomes damaged in people who tend to oscillate primarily between the first two systems, that is, between aversion (the threat system which operates through fight, flight, or freeze responses to perceived social or other threats) and striving (the drive system which is seen in the brains' efforts to address the threat; Gilbert et al., 2009). This type of brain activity can draw people into loops of brooding, rumination, and (particularly anticipatory) worry, and therefore a striving to address the perceived threat(s). The purpose of such striving is to avoid inferiority (Gilbert et al., 2009), which has been studied by the Compassionate Mind Foundation among UK university students.

Similarly, provided that they can be motivated to practice social connectivity through developing compassionate communication abilities in the context of online group meetings, which in turn facilitates stimulating their soothing systems, Sri Lankan students were a viable sample for an intervention based on compassionate communications for online group work management.

## 2. Materials and methods

In this action research study, STEM students studying at five Sri Lankan state universities participated in the study online. The study comprised six student groups, each one consisting of four students who were pursuing their STEM-related degree programmes. Each group comprised a combination of Sinhalese, Tamil, and Muslim students and representation of both genders and up to four Sri Lankan universities. The latter was to ensure the participating students were likely strangers to each other. The focus on STEM students was in keeping with the World Bank's funding of Sri Lanka in the education of, specifically, its STEM students in emotionally intelligent communication skills (AHEAD, 2018).

### 2.1. The aim of the current study

The primary research question is as follows: Can Sri Lankan-based STEM students be motivated to switch their cameras on during their online group work meetings by learning about the cognitive skills of compassionate communication? The sub-research questions were as follows:

a) Is there a significant difference in the screen gaze behaviors of the respondents before and after the CSCC intervention? Based on this, the following hypotheses were developed:
  H_0_: There is no difference in students' screen gaze behaviors that could be attributed to the CSCC intervention.
  H_1_: There is a difference in students' screen gaze behaviors that could be attributed to the CSCC intervention.
b) Are there any observable changes in respondents' behaviors during their group work meetings before and after the CSCC training intervention?
c) How do respondents perceive the application of common shared virtual background in their online group work meetings?

In particular, the first sub-research question is answered through quantitative data analysis, the second sub-question is addressed through both quantitative and qualitative data analysis, and the third sub-question is addressed through qualitative data analysis.

### 2.2. Sampling

To recruit the sample from the population (*n* = 36, 388) for this study, a poster designed explaining the project was emailed, together with a volunteer participants registration link, to all Deans of the Faculties and/or Heads of Departments in five (*n* = 5) Sri Lankan universities. All of them agreed to circulate the poster and the registration link among their 2nd-year STEM students. After volunteers had registered via the link, the necessary strata (gender and membership of either the Sinhalese, Tamil, and Muslim groups in SL) were identified and their status as STEM students was checked. Stratified sampling (Thomas, 2020) was combined with maximum variation sampling (Cohen and Crabtree, 2006). This was so that each group of four students (six groups in total, *n* = 24) comprised a balanced representation of all target groups: Muslim, Sinhalese, and Tamil. Moreover, there were two male and two female students allocated to each group for a gender balance. All groups comprised four students who were each from a different Sri Lankan state university to better ensure that there were no already established friendships in play. Overall, the aim was and so to reflect the diversity of the target population (Thomas, 2020).

### 2.3. Data collection

A mixed-methods approach was used to collect and analyse data. Primary data were collected using five tools: video recordings of all the group work meetings,^1^ focus groups/interviews,^2^ ethnographic field notes, and two questionnaires, i.e., a questionnaire on previous group work experiences (Gilbert, 2016; University of Hertfordshire, 2020), and the Compassionate Mind Foundation's Compassionate Engagement and Action Scale. Both questionnaires were administered before and after the CSCC intervention. The overall procedure comprised three phases as explained next.

#### 2.3.1. The pre-intervention task-focused meeting

First, each of the six groups of *n* = 4 students per group participated in a video-recorded (control) task-focused group discussion as follows. Each student presented a self-chosen research article related to their STEM field and then the whole group discussed it. In other words, this procedure was repeated for all four students in each group for all six groups.

Then, students participated in a video-recorded focus group and/or semi-structured interviews to explain their lived group work experiences of this meeting (the control). They were invited to share their past lived experiences of any previous online task-focused group work meetings they had participated in, including as a result of the pandemic.

Furthermore, students filled in the two above questionnaires provided online (see these in Supplementary material 1, 2). The data from this first use of the two questionnaires were kept for comparison with data from the second iteration of the two questionnaires after the second task-focused presentation and discussion team meeting and follow-up focus group (after the intervention).

#### 2.3.2. Intervention

The Cognitive Skills of Compassionate Communications (CSCC) for task-focused group work meetings were taught to students through a 90-min intervention session. During this interactive intervention session, the key theory of compassion in terms of brain function was introduced to the group members. This included an explanation of the psychobiological model of compassion (Compassionate Mind Foundation) so that students could understand the science-based rationales by using the following practical strategies of CSCC that were introduced next to help students demonstrate their full attention to all others in the group.

##### 2.3.2.1. Non-verbal examples

When others speak: Nodding, encouraging (e.g., thumbs up and smiling) including to show agreement and/or understanding; or, indicating lack of understanding, e.g., by facial expression and/or hand waving; or else waving to call for a turn without interrupting (digital or physical hand waves).

*NB:* Thus, it becomes clear to students why the support of each other's psychological safety, as others protect theirs, requires camera use across the group.

Allowance of reasonable silence to let the group think and process what has been said so far without jumping straight in at the expense of (shyer and/or international) students hoping to speak.

##### 2.3.2.2. Verbal examples

Using warm voice tone and group members' names to:

Intervene in non-contributing behaviors by inviting the quieter student(s) to add their view if they would like to. All other members are equally responsible for offering these opportunities *in this way*, during the meeting.
If/when a speaker “freezes” (c.f. threat system activation), others may prompt to help, not opportunistically, take over the talk. Or, the speaker in difficulty may ask another for help, e.g., “could you help me out here please, Ahmed?”
Intervene in monopolizing behavior, by *validating* the monopoliser for a (“useful”/”relevant”/”crucial”/”helpful”/”key”/”interesting”) point just made and why it was so (before e.g., going on to invite another to speak, as above).
Thanking/complimenting others for their contributions; with reasons where useful, critically.
Demonstrating in the discussion that close attention was paid to each speaker, e.g., through *relevant* responses, such as questions, points, perspectives, and/or ideas.

The above examples are the key, evidence-based features of compassionate communication strategies (for team meetings) offered during the intervention 90 min intervention workshop.

##### 2.3.2.3. Interactive aspect of the workshop

To assist in the learning of these, the interactive component of the workshop included:

Inviting students to explain a group work that had taken part in so the others to discuss, so that everyone had the chance to put into practice the above skills. The instructor attempted to disrupt the flow of shared talk as a monopoliser and then as a non-contributor for the group members to employ the above compassionate strategies to address those behaviors effectively.

Overall, the intervention sought to develop explicitly the deployment and recognition (of others') skills at dismantling monopolizing/dominating behaviors, including by non-verbal means, without silencing anyone. It was, further, to address non-contributing behaviors, again using a warm voice tone, name, and also critical thinking to invite quieter students frequently into mindfully created safe spaces to contribute to the group discussion. The teacher/trainer was always the same for each group. The CSCC training session was conducted in the English language.

#### 2.3.3. Post-intervention

After the CSCC session, the same groups of four students conducted a task-focused group work meeting online—this time bringing a *new* self-chosen article. The group decided on the order of presenting their journal articles and inviting the first group member to present their self-chosen article, and then others for discussing the content of the article. This repeats until all four members present their articles and all four members contribute to each discussion.

Then, each student filled in the same two questionnaires they had completed for the pre-intervention so that results between pre- and post-intervention could be compared. Next, the students participated in a new focus group and/or interviews to explain their lived learning and social experiences of this second group meeting [in comparison with (i) the control discussion above in which they had participated].

These focus groups/interviews were conducted after each group meeting (pre- and post-intervention) to collect responses to each group meeting experience from the respondents. This enabled the exploration of any changes in responses to the post-intervention meeting compared to the pre-intervention meeting. It should be noted that none of the participants was a native English speaker and that all names used to refer to them below are pseudonyms.

### 2.4. Application of shared virtual background

As this was action research (Lewin, 1946; Kemmis and McTaggart, 1982), after understanding the possible negative impacts of background distractions during the pilot study,^3^ the potential use of shared virtual backgrounds was explored with all participants as an amendment to the initial research design.

i This could create virtual, visual boundaries around each student within a single commonly experienced background/environment. This would limit the visual fields so that no group member would be visually aware of the presence of anyone outside the group.
ii Exaggerated body movements, e.g., turning away to communicate outside the group, would be highlighted to the whole group because the student would likely completely disappear from the screen.

### 2.5. Data analysis—Quantitative data

The quantitative data were collected from the pre- and post-intervention task-focused group work meetings and the two questionnaires.

#### 2.5.1. Screen gaze behaviors of the group members

Screen gaze behavior data of the group members (during the pre-and post-intervention task-focused group work meetings) were quantitatively analyzed using three tools: Wilcoxon Signed-Rank Test (King and Eckersley, 2019) using R, and plots created using R and MS Excel. These were applied to data derived from second-by-second analysis of every group member's video-recorded screen gaze behaviors, i.e., during every presentation and every group discussion during both the pre- and post-intervention task-focused group work meetings. The data were then entered into R to perform the Wilcoxon Signed-Rank Test to explore whether there was any statistically significant difference between the screen gaze behavior of the group members before and after the CSCC intervention. For the next stage of analysis, R plots were created to identify and compare group members' screen gaze behaviors individually, according to their real-time roles, that is, when they were (a) presenting their second chosen journal article, (b) listening to others presenting, or (c) discussants during the group meetings. Finally, the overall percentage of each group's screen gaze data before (pre-intervention) and after the CSCC intervention session (post-intervention) were used to generate graphical illustrations of the groups' results during each presentation and each follow-up discussion through MS Excel scatter charts.

### 2.6. Data analysis—Qualitative data

The overarching approach taken to identify key themes arising from the transcriptions of group work meetings and focus groups/interviews was Template Analysis (TA; King, 1998, 2004; Brooks et al., 2015). To support the use of TA, NVivo (Pro 12) was used to code the data.

#### 2.6.1. Analysis of pre- and post-intervention group work meetings transcriptions

Transcriptions of all pre-and post-intervention group work meetings and focus groups were uploaded into the NVivo (Pro 12) for analysis. The *n* = 12 transcriptions (i.e., for six groups before the intervention, and then again afterward) were repeatedly trawled for codes that might otherwise be missed, and this also allowed constant cross-coding within each of the identified themes. In coding data, first free codes (grouping similar words, phrases, and meanings) were identified. Then, focused (interpretive) codes (grouping the codes that convey similar meanings or contribute to constructing a single argument) were identified to derive interpretive meanings (King and Horrocks, 2010). As the third and final step in the data coding, it was possible to identify what the emergent overarching themes were.

Video recordings of task-focused group meetings were made while student groups conducted their pre- and post-intervention group meetings. This is in keeping with extant research on optimal task-focused, online discussion group size. Transcriptions of pre- and post-intervention group work meetings were analyzed separately by applying TA. To identify the themes, the coding of data was carried out in the same manner as outlined above. Next, the themes that emerged from the pre-intervention group meetings were compared with the emergent themes from the post-intervention group meetings.

#### 2.6.2. Analysis of pre-and post-intervention focus group/interview transcriptions

The focus groups/interviews conducted after each pre- and post-intervention group meeting were also video recorded and transcribed. All focus group/interview transcriptions were uploaded into NVivo (Pro 12), and the data were coded using the same procedure as above. Then, the themes that emerged from the pre-intervention focus groups/interviews were compared with the emergent themes from the post-intervention focus groups/interviews.

#### 2.6.3. Micro-ethnographic analysis

In addition, a close analysis of the video-recorded student behaviors was carried out using McDermott's (1988) micro-ethnographic methods for analyzing filmed classroom behaviors. Specifically, in this study, McDermott's methods were used to analyse the behaviors of each respondent in their meetings before and after their training in CSCC.

The second-by-second micro-ethnographic analysis was conducted to identify changes in time spent by respondents' on-screen gaze time attentiveness to others during both pre- and then post-intervention. It also allowed close observation of changes in facial expressions and the mobility of these changes as students responded to each other. During this analysis, particular themes that appeared most aligned with the group's overall behaviors could be identified and compared. Any critical incidents (interactions of note), how they occurred, and how they were responded to throughout the unfolding interactions in the group were viewed repeatedly for close analysis via the video footage.

Then, the results of these both pre-and post-intervention qualitative analyses were compared to explore differences, if any, in individual and/or group behaviors after the CSCC intervention session. The analytical findings here were compared closely with other data sets, for example, student-reported critical incidents around their communicative ease or otherwise in the task-focused meetings, that they talked about during the focus group meetings. All results in the study were triangulated.

## 3. Results

The results of the current study indicate the practicality of developing cognitive skills of compassionate communication among Sri Lankan HE STEM students in their online group work meetings. These findings should be seen in relation to students' reports (in their first focus groups) of their lived experiences of their HE online group/teamwork before this study. The following examples were typical of what students described across all the groups, namely that monopolizing behavior by one or more students in these meetings had been common, as had non-contributing behaviors. Overall, it was felt that students had not shared equal time during their group meetings.

**S10:** *Definitely not. Some people are speaking a lot of time. Some guys speak less. Sometimes, some guys are not speaking* (Group 1, Transcription of Pre-intervention, Focus Group, p. 5, lines 121–122).
**S18:** *We always hearing hearing hearing. But in this time* [during this study], *we are talking* (Group 3, Transcription of Pre-intervention, Focus Group, p. 4, lines 118–119).

There was also overall agreement that it was usual for most and sometimes all students to keep their cameras switched off.

**S12:** *This is the first time for me [switching on the camera]. So, it was a bit of nervous…* (Group 1, Transcription of Pre-intervention, Focus Group, p. 13, lines 332–333).
**S15:** …* really [we] don't like to switch on the cameras because we are from a different place, … and sometimes, the backgrounds, they are not much good* (Group 1, Transcription of Pre-intervention, Focus Group, p. 17–18, lines 492–494).
**S17:** *I don't I feel a bit more at ease when the camera is switched on* (Group 1, Transcription of Pre-intervention, Focus Group, p. 12, lines 397–398).

A comparison of the (pre- and post-CSCC interventions) quantitative and qualitative results revealed a significant increase in group members' social and learning experiences and how the first mediated the second. The findings illustrate how the CSCC training resulted in group members turning their cameras which in turn led to an increase in sustained and attentive screen gaze during the post-intervention online group meetings. The results also shed light on why students switched their cameras on after learning how and why screen gaze supported others in the group, including strangers, as illustrated in the data presented below.

### 3.1. Comparison of group members' screen gaze behaviors before and after the CSCC intervention

To compare and contrast screen gaze and related behaviors of respondents, before and after the CSCC intervention, this section presents the results of three quantitative analyses for how the results of each might (or might not) inform each other.

#### 3.1.1. The Wilcoxon Signed-Rank Test to compare screen gaze behaviors of group members as to the roles they perform in groups before and after the intervention

The Wilcoxon Signed-Rank Test was run in R to identify whether there was any difference in the screen gaze behaviors of the group members before and after the intervention. As explained in Section 2.4 on the quantitative data analysis, *p*-values have been calculated to quantify the impact of the intervention on different types of respondents, namely presenters, listeners, and discussants. Table 1 shows the results for all six groups. There are four members under each type of respondent in each group.

**Table 1:** The Wilcoxon Signed-Rank Test *p*-value results for screen gaze behaviors of group members as to the roles they perform in the groups.

**Group number**	**Types of respondents**		
	**Presenters**	**Listeners**	**Discussants**
Group 1	0.125	0.001953	0.0002441
Group 2	0.0625	0.002961	1.526e-05
Group 3	0.0625	0.009766	3.052e-05
Group 4	0.125	0.0002441	1.526e-05
Group 5	0.1875	0.001709	0.0001526
Group 6	0.0625	0.0002441	0.0002407

As indicated in Table 1, the Wilcoxon Signed-Rank Test results for screen gaze timing data in pre- and post-intervention for each group *p* < 0.05 indicated that there was a significant difference in the gaze behaviors of group members after the CSCC intervention session.

##### 3.1.1.1. Presenters

The percentage screen gaze of all the presenters was considered independently for each group. The *p*-values for the presenters as shown in Column 2, Table 1 revealed an increase in sustained screen gaze with all the 12 presenters after the CSCC intervention session. However, as indicated by the relatively high *p*-values, the increase was found to be not statistically significant in the presenters.

##### 3.1.1.2. Listeners (presenter's audience members)

As shown in column 3 of Table 1, there was a statistically significant increase in sustained screen gaze of those listening to the presenters after the CSCC intervention session. For all six groups, the *p*-value is < 0.01, meaning the probability of the null hypothesis being true is <1%. Hence, the null hypothesis (H_0_) that says there is no difference between the screen gaze behaviors of the group members before and after the CSCC should be rejected and the alternative hypothesis H_1_ was accepted for the listeners.

##### 3.1.1.3. Discussants (screen gaze behaviors during the discussion component of the group work)

As shown in column 3 of Table 1, there is a statistically significant increase in sustained screen gaze of the group members when they perform as discussants during the follow-up discussions after the CSCC intervention. Therefore, the null hypothesis (H_0_) should be rejected and the alternative hypothesis H_1_ was accepted by the discussants.

#### 3.1.2. R plots to compare individual group members' screen gaze behaviors before and after the intervention

**Figure 2:**
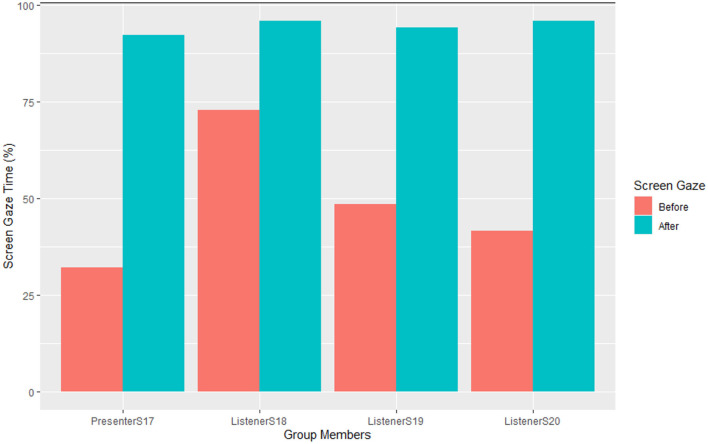
Group members' screen gaze during S17′s pre- vs. post-intervention journal article presentations.

iR plots were created to analyse and show graphically the screen gaze behaviors of each individual group member during each presentation. Then, more R plots were created separately to show the screen gaze behaviors of individual group members during every follow-up discussion. Figure 2 illustrates group members' percentage screen gaze during their pre- vs. post-intervention discussions of S17′s two self-chosen journal articles.

S17 was the first presenter in group 3 of six groups (so the second of the two mid-groups). She is a useful starting point for the example shown below, and her data and that of others in her group in all their roles as well (as hers) were very similar to that of all other groups (full data sets available). In this example, the orange bars represent the percentage screen gaze of individual group members before the CSCC intervention session (pre-intervention). The turquoise bars represent the percentage screen gaze of the group members after the CSCC intervention session (post-intervention).

Figure 2 shows that before the CSCC intervention session, S17 (while presenting her article) sustained her screen gaze for 32.04% of the time while S18, S19, and S20 (her listeners) sustained their screen gaze for 72.89, 48.59, and 41.55% of S17′s presentation time, respectively. In contrast, after the CSCC intervention session, S17 (the presenter) sustained her screen gaze for most (92.34%) of her journal article presentation. Similarly, listener S18 sustained his screen gaze for most of the whole of S17′s presentation (up from 32.04% for S17′s previous presentation to 95.95%); listener S19 sustained his screen gaze for 94.14% (up from 48.59% for S17′s previous presentation) and S20 sustained her screen gaze for 95.95% (up from 41.55% for S17′s previous presentation). These results were found to be representative of all presenters' sustained screen gaze behaviors and that of their listeners.

**Figure 3:**
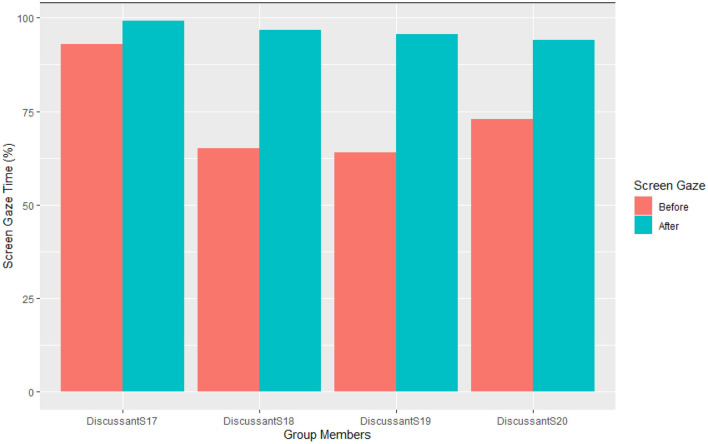
Group members' screen gaze during S17′s pre vs. post–intervention journal article presentations.

Next, Figure 3 illustrates each of Group 2′s members' percentage screen gaze during their pre- and post-intervention follow-up group discussions of S17′s two self-chosen journal articles.

Figure 3 shows that before the CSCC intervention session, during the follow-up discussion of S17′s journal article, the discussants: S17, S18, S19, and S20 sustained their screen gaze attentiveness for 92.99, 64.97, 64.01, and 72.93% of the discussion time, respectively. In contrast, after the CSCC intervention session, S17 sustained her screen gaze through almost the whole of the discussion (99.20%). S18, S19, and S20 increased their screen gaze substantially (96.79, 95.58, and 93.98%, respectively) compared to their pre-intervention discussion in S17′s article.

Overall, taking together the journal article presentations and follow-up discussions shows a substantial increase in sustained scree gaze of all group members after the CSCC intervention session.

Next, the pre- and post-intervention results of the whole group's average screen gaze during each group's presentations and follow-up discussions were explored through MS Excel.

#### 3.1.3. Microsoft excel analysis—Group vice screen gaze before and after the intervention

**Figure 4:**
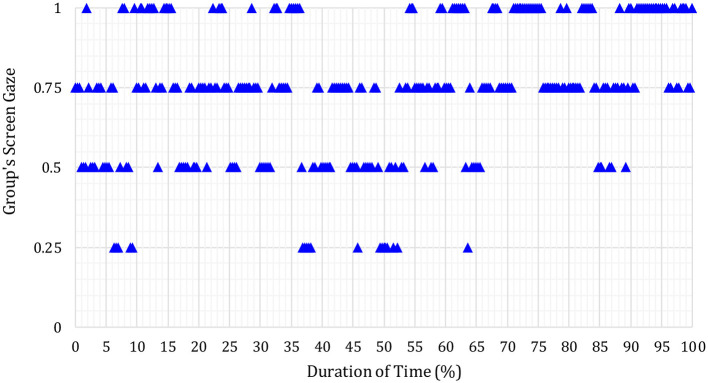
Whole group's screen gaze during discussion on S17′s journal article (post-intervention).

**Figure 5:**
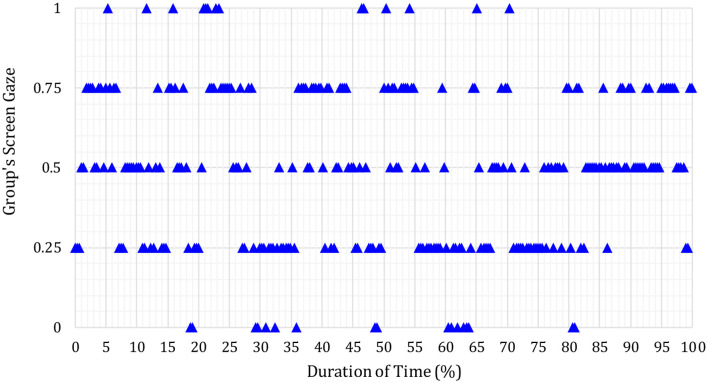
Whole group's screen gaze during S17′s pre-intervention journal article presentation.

**Figure 6:**
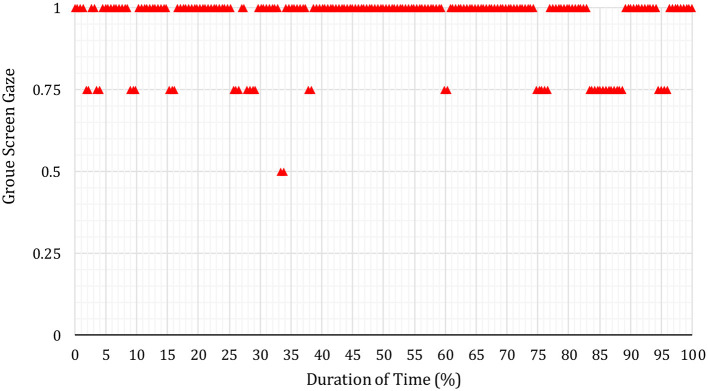
Whole group's screen gaze during S17′s journal article presentation (post-intervention).

In Figures 3–**6** below, the Y-axis indicates each group's average screen gaze values are as follows.

- 0 = no one (0%) offers screen gaze at any time in the meeting.
- 0.25 = only one group member (25%) offers screen gaze.
- 0.5 = two members of the group (50%) offer sustained screen gaze.
- 0.75 = three members of the group (75%) offer screen gaze.
- 1 = all four members (100%) offer screen gaze.

It was found overall that the example results below were representative of participants' pre- and post-intervention screen gaze behaviors across all groups.

The blue triangles represent the screen gaze of the whole group during S17′s first (before the intervention) journal article presentation.

As shown in Figure 4, blue triangles majorly on 0.5 screen gaze attention level show that only two group members sustained screen gaze during most of S17′s presentation before the intervention. As can be seen in Figure 4, all four members sustained screen gaze together (i.e., at the same time) only on a few occasions (4.96% of the time duration of the presentation).

Next, in Figure 5, red triangles represent the sustained screen gaze of the whole group during S17′s second (after the intervention) journal article presentation.

In Figure 5, most of the red triangles are on the 1 (one) screen gaze attention level. This shows that all four group members sustained screen gaze during most of (79.82% of total time duration) S17′s presentation after the intervention. When compared with the pre-intervention (blue triangles) in Figure 4, respondents were found to sustain screen gaze notably more time in the post-intervention (red triangles) in Figure 5 in relation to the S17′s journal article presentation.

These results are representative of what was also found for other groups. Figure 6 in the example below shows the average screen gaze of the whole of the group during the follow-up discussion after S17′s first (i.e., pre-intervention) journal article.

The blue triangles seen at the 0.5 and 0.75 screen gaze attention levels, respectively, indicate that only two or three members sustained screen gaze together at any time during the whole group's follow-up discussion of S17′s journal article before the intervention. All four members sustained screen gaze together (i.e., at the same time) only for a few occasions.

**Figure 7:**
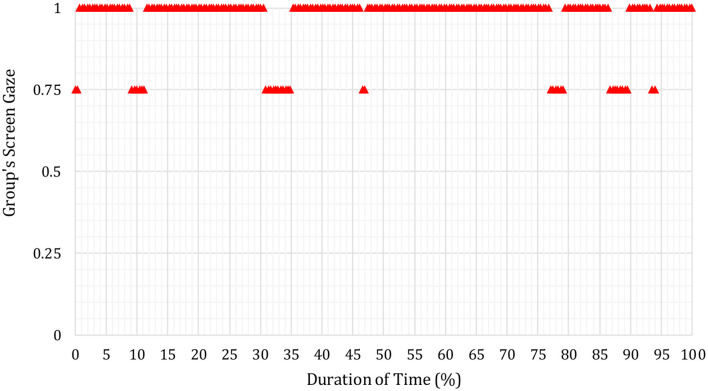
Whole group's screen gaze during discussion on S17′s journal article (pre-intervention).

Figure 7 below shows the screen gaze of the whole of the group during the follow-up discussion of S17′s second (i.e., post-intervention) journal article presentation.

From the number of red triangles that are at the screen gaze attention level 1 (one), it can be seen that all four members of the group including S17 (i.e., 100% of the group) sustained screen gaze throughout almost all of the discussion on S17′s second journal article presentation after the CSCC intervention session. This result was consistent with all groups and therefore representative of the results from all groups.

Overall, the Microsoft Excel analysis results of all groups showed a substantial increase in all groups' average screen gaze after the CSCC intervention session. In terms of triangulation, these MS Excel results offered a different comparative perspective on the group members' pre- and post-intervention screen gaze behaviors, but the results corroborated the Wilcoxon Signed-Rank Test (see Section 3.1.1) results, and the results too from the plots created through R programming language (see Section 3.1.2).

Importantly, all of the quantitative results so far do not mean that the CSCC can be regarded as the only causal factor for the behavioral changes seen in these tests. Perhaps the fact that the students were meeting again and becoming more familiar with each other was part of the reason for the quite rapid change in respondents' screen gaze behaviors.

Therefore, next, to explore what might, or might not, have contributed to the rapid changes in screen gaze behaviors identified above, the results of the micro-ethnographic (micro-observational) data analysis are explored, which informed the above results. First, a summary of the results found through micro-ethnographic analysis of the pre- and post-intervention group behaviors of the respondents is presented.

#### 3.1.4. Micro-ethnographic analysis

Micro-ethnographic field notes of the pre- and the post-intervention group meetings were analyzed, and the results were compared to explore for any differences/similarities in respondents' behaviors before and after the CSCC intervention session.

Here is an example from the pre-intervention screen gaze behavior of S17 during her presentation to her fellow group members. The behaviors seen in Box 1 and Box 2 below were characteristic, pre-intervention, of all the participants in all groups when they were presenting to the rest of the group. That is to say, the results for pre-intervention screen gaze attentiveness during the task-focused meetings were found to be similarly fragmented, erratic, and unpredictable across all participants in both UK groups irrespective of close examinations through the videos for disconfirming evidence of this from any group member.

Box 1 **Micro-ethnographic field notes during S17′s journal article presentation (pre-intervention).**

**Group 3/pre-intervention screen gaze and related behaviors of group members during S17′s presentation**

*Total time duration: 4 min and 44 s*.

1. S17 breaks screen gaze for a combined total of 3 min and 13 s with the group during her presentation.
2. The presenter appears to be reading much of the time with her head down. she looks to be breaking the optimum gaze without maintaining eye gaze with other group members for most of her presentation, she presents while looking at her notes. She only occasionally and briefly connects with other group members through screen gaze.
3. After this, S18 looks to his right and it appears, he does something with his hands while non-verbally communicating with another person outside their group. Afterward, he fixes his gaze downward and time to time looks to his right, breaking his screen gaze for a combined total of 1 min and 17 s during S17′s presentation.
4. S19 also looks to his left and up most of the time while touching his chin and scratching his nose with his hands, and then he fixes his gaze downward and after that circles his eyes around breaking screen gaze for a combined total of 2 min and 26 s and seldom looks at the screen.
5. S20 also looks to her right and time to time turns off her webcam. After that, she continuously looks down breaking screen gaze for a combined total of 2 min and 46 s.

Box 2 **Micro-ethnographic field notes during S17′s presentation (post-intervention).**

**Group 3/post-intervention screen gaze behavior of group members during S17′s journal article presentation**

*Total time duration: 3 min and 42 s*.

1. S17 breaks screen gaze for a combined total of 17 s, and she sustains screen gaze during most of her presentation.
2. S18 breaks his screen gaze for a combined total of 9 s but otherwise sustains his screen gaze during the rest of S17′s presentation.
3. S19 breaks screen gaze for a combined total of 13 s and all the other time he sustains screen gaze.
4. S20 breaks the screen gaze only for 9 s during S17′s presentation and all the other times she sustains the screen gaze.

Notably, the results on screen gaze behaviors of group members during the pre-intervention were found to be erratic and unpredictable across all participants. This again was despite the close search through the videos for disconfirming evidence of this by any group member. Overall, it was found that screen gaze was better sustained across the groups after the CSCC intervention session as shown in Box 3 and Box 4. These data inform and appear to corroborate what was found in the quantitative data above in Sections 3.1.1, 3.1.2, and 3.1.3 regarding screen gaze. Importantly, these micro-ethnographic field note findings were found to be representative of all groups.

Box 3 **Micro-ethnographic field notes during the discussion of S17′s journal article (pre-intervention).**

**Group 3/pre-intervention screen gaze behavior of group members during the discussion on the journal article presented by S17**.

*Total discussion time: 5 min and 14 s*.

1. During the discussion of the journal article presented by S17 for 5 min and 14 s, S17 breaks screen gaze for a combined total of 22 s.
2. S18 breaks screen gaze (looks downward, left side, and looks away) for a combined total of 1 min and 50 s. •S19 breaks screen gaze for a combined total of 1 min and 53 s as he looks away.
3. S20 also breaks screen gaze for a combined total of 1 min and 15 s during this discussion as she looks down and then she turns off her webcam for the rest of the discussion.

Box 4 **Micro-ethnographic field notes during the discussion of S17′s journal article (post-intervention).**

**Group 3/post-intervention screen gaze behavior of group members during the discussion of the article presented by S17**.

*Total discussion time: 5 min and 14 s*.

1. After S17 presents her journal article, the group discussion of it lasts for 4 min and 9 s. S17 sustains screen gaze throughout the whole discussion time, except for a 2 s break in her screen gaze.
2. S18 breaks screen gaze for a combined total of 8 s, and he sustains screen gaze throughout all the other discussion time.
3. S19 breaks screen gaze only for a combined total of 11 s. He sustains screen gaze during most of the discussion of S17′s journal article.
4. S20 breaks screen gaze for a combined total of 15 s and she sustains screen gaze during most of the discussion of S17′s journal article.

Next, the emergence of screen gaze as a theme through students' accounts for the change in their screen gaze behaviors is discussed with the examples extracted from the transcriptions of focus groups.

#### 3.1.5. Template analysis of the focus group transcripts (NVivo Pro 12): turning cameras on/off

In the pre-intervention focus groups, students reported a common reluctance—their own and others'—to turn cameras on (even if they could) during their previous group meetings on their programmes and not just during the pre-intervention group meetings of this study. Again, social anxiety appeared to be the main reason that students described feelings of unease about their personal appearances and/or the appearance of their personal physical backgrounds (e.g., of their rented rooms), seeing that people including strangers were watching them, the belief that they could talk more confidently with the camera switched off and even engaging in other work at the same time as their group meetings were taking place. Example extracts from focus group transcripts are presented next.

**S10:** *If we switch off the camera we can talk confidently more than switch on the camera. … Because sometimes friends [were] watching me. That is very excited to me [sic], very nervous because everyone is watching me* (Group 1, Transcription of Pre-intervention, Focus Group, p. 13, lines 341–342).
**S22:** …* we usually don't like switch on the camera and switch on the microphone as well … if we have anything to ask, on that time only we unmute and ask, and the other times we usually don't do*. … *this is my first ever experience I had switched on it [camera]* (Group 1, Transcription of Pre-intervention, Focus Group, p. 11, lines 373–377).

In contrast, the general reluctance (across all groups) to switch on cameras was found to be reduced after the CSCC intervention as students stated that they preferred to speak with their cameras on in online group meetings during the post-intervention focus groups, as here:

**S10:** …* definitely it was changed, before I usually speak without camera but now I am comfortable with camera [switched on]* (Group 1, Transcription of Post-intervention, Focus Group, p. 8, lines 246–247).
**S11:** *Before this meeting I felt, switch off the camera and speak. And now I am okay, I got confident from this [CSCC]* (Group 1, Transcription of Post-intervention, Focus Group, p. 12, lines 313–314).

During the post-intervention focus groups, respondents pointed to their practical use of CSCC as a key factor in turning on their cameras (in contrast to their past practice), and also in sustaining screen gaze attentiveness to their group members online. This student-reported motivation to turn their cameras on appeared to have not only arisen because cameras facilitated group members' observations (noticing) of their own and others' non-verbal communications as a means to demonstrate validation of others' efforts but also, if someone needed help in understanding or encouragement to speak or continue speaking, others could now see this requirement if they paid attention (i.e., “noticed” c.f. the definition of compassion). Hence, this ability in the teams to “notice” appeared to help them develop their own compassionate strategies, e.g., circumlocution (rephrasing of some points) for their fellow non-native English speakers, so that no one should be disadvantaged because of lower levels of English understanding (e.g., as related to different socio-economic backgrounds). Here are more examples of students' purposeful micro-observations of each other.

##### 3.1.5.1. Presenting

**S13:** …* when I was presenting my research article, I saw facial expressions of the others. I saw that Shivani, she listened well. I think Shenab listened well, and she also could get something. So, I can get something from their facial expressions. That is how it helps* (Group 2, Transcription of Post-intervention, Focus Group, p. 6 and 7, lines 162–165).
**S18:** … *while I am [was] continuing to present, the presentation, my other group members are [were] nodding head and appreciating, so, those supported continuing my presentation. It's a credit for my presentation in a good way and very helpful to me* (Group 3, Transcription of Post-intervention, Focus Group, p. 2, lines 52–55).
**S19:** *When presenting, giving our facial expressions, the facial expressions motivate them* (Group 3, Transcription of Post-intervention, Focus Group, p. 5, lines 164–165).
**S34:** *Switching on the camera and talking [is better], because we can see their reactions, whether they understood or not* (Group 6, Transcription of Post-intervention, Focus Group, p. 15, lines 515–516).

##### 3.1.5.2. Listening

**S13:** *...during the discussion we used eye contact and also really, I try to listen very well* (Group 2, Transcription of Post-intervention, Focus Group, p. 3 and 4, lines 75–78).
**S20:** *When they speak, we show our reactions for them to engage* (Group 3, Transcription of Post-intervention, Focus Group, p. 5, lines 140–142).
**S34:** …* we noticed each and everyone's facial expressions and also they showed, whether they understood or not* (Group 6, Transcription of Post-intervention, Focus Group, p. 7, lines 218–220).

##### 3.1.5.3. Discussing

**S20:** *When we talk, when others talk, we observe others and also observe our facial reactions and, in our face, [facial] reactions we make comfortable, the group discussion we know how to attract or interact discussion with others* (Group 3, Transcription of Post-intervention, Focus Group, p. 2, lines 34–36).
**S17:** *I think it helps learning because, when we see that from their reactions, like nodding of the heads, we know that they understood what we are saying* (Group 3, Transcription of Post-intervention, Focus Group, p. 5, lines 145–146).
**S30:** *I think, when we use them, compassionate strategies, it can motivate people. … we can be much more understanding of the other person or our team members. So, that will definitely motivate the group. And why we need motivation? because I mean no one wants to be in a team that is really weird. I don't want to be in a team, if the other team members don't understand me, when they're not compassionate with me, so I think when we use the compassionate strategies in teams, it motivates other people, there by promoting healthy work experience within the group* (Group 5, Transcription of Post-intervention, Focus Group, p. 5, lines 145–146).

These themes were not evidenced during the pre-intervention group meetings. That is to say, the CCSC appears to have channeled students' closer attention to their own and each other's non-verbal communications and the significance of these for communicative ease in the group.

Overall, analysis of the pre-intervention focus groups transcripts in Cycles 2 and 3 revealed that levels of psychological safety were not optimal due to social anxieties which the students explained above. In contrast, in the post-intervention focus group transcripts, students reported reductions in their anxiety. This may also explain how the groups achieved a more equalized level of agency, or participation, during their post-intervention group meetings. This means that social efforts to help others contribute led to better group learning. Overall, the post-intervention discussions were longer than the pre-intervention discussions. The latter were also critically richer with students offering more explanations of the points they wished to make and/or offering examples for discussion.

### 3.2. Application of a shared virtual background

**Figure 8:**
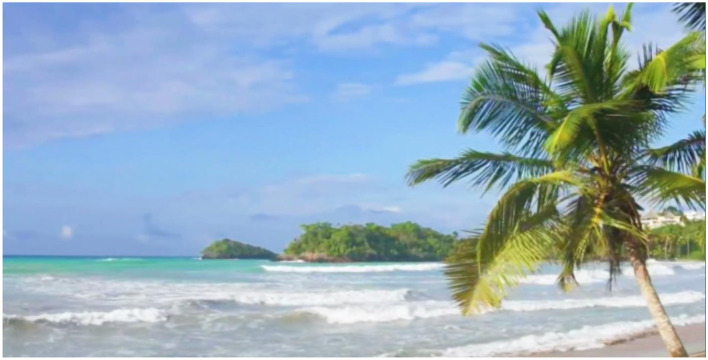
Virtual background: the Caribbean Sea with moving waves and waving leaves of a palm tree. This virtual background was obtained from the Zoom video conferencing platform.

**Figure 9:**
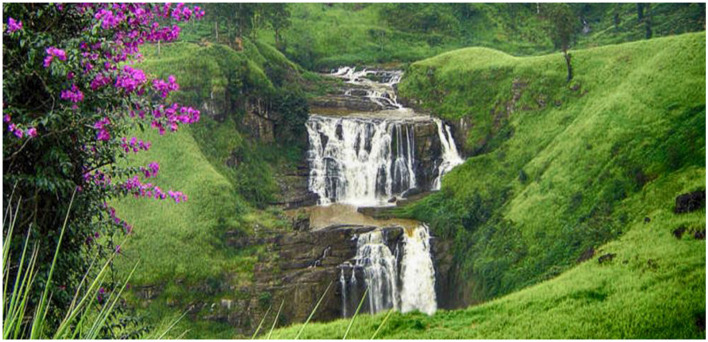
Virtual background: Sri Lankan greenery hillside with St. Clair fall. This image was obtained from: https://www.srilankatailormade.com/rainbow-tour-in-sri-lanka/.

**Figure 10:**
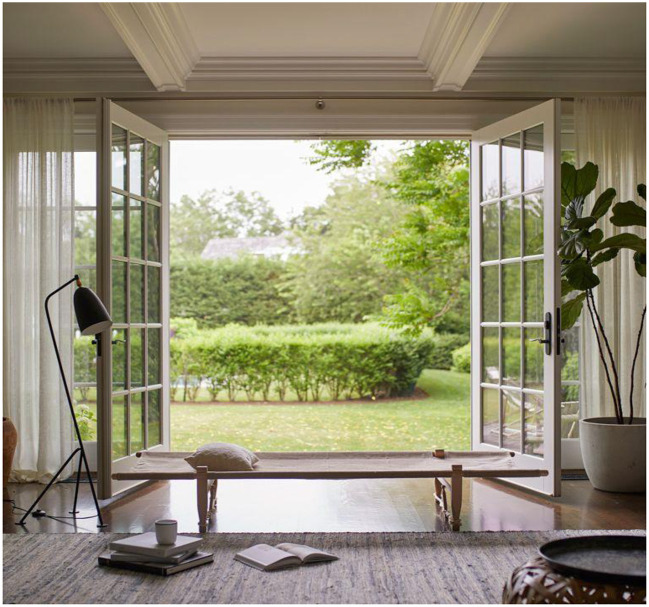
Virtual background: opened window to a green environment (design by Melissa Lee of bespoke only; photo by T. Y. Cole). This virtual background was obtained from: https://www.mydomaine.com/interior-design-zoom-backgrounds-4842797.

Many background distractions/activities were going on sometimes in students' home environments. Therefore, each group was asked if they might like to choose a virtual background that they could share to reduce the effect of such distractions. One group opted for the zoom background immediately below (Figure 8). The other five groups asked for a selection to be provided, and so in line with the beach background here, a selection of 10 was offered that also drew on natural surroundings and could be considered soothing in line with the three circles model of the Compassionate Mind Foundation (see Figure 1, above). Three out of ten of these images were of natural and authentic featured backgrounds from Sri Lankan nature. Three groups opted for one of these three (see Figure 9 below). The other two groups chose the image in Figure 10, below.

#### 3.2.1. Analysis of focus group transcriptions

This part of the analysis discovered positive insights from the group members on why applying a shared, soothing virtual background so that each group as a whole appeared to be together in one nature-themed place seemed helpful to them.

**S9:** *This virtual background is relaxing and is better than the normal background and it's seen if someone is distracted from the group meeting as the whole body is disappeared from the screen. Thus, we are motivated to focus attention and to be on screen* (Group 1, Transcription of Post-intervention, Focus Group, p. 16, lines 499–502).
**S13:** *In online meetings, we can make a peaceful mind by sharing same background* (Group 2, Transcription of Post-intervention, Student Comments, p. 1, lines 27–28).

Feelings arising from improved group cohesion were also noticed, as evidenced in these student remarks:

**S17:** *And you also get a feeling of sense of belongingness, because you have that same background* (Group 3, Transcription of Post-intervention, Focus Group, p. 14, lines 499–500).
**S35:** *By sharing this same background, I can feel everyone is at the same place. We are somehow we are at different places, but the background makes all our thoughts into one place* (Group 6, Transcription of Post-intervention, Focus Group, p. 13, lines 449–451).

These accounts suggest a good level of psychological safety, one of the key values of a shared commitment to compassion within team dynamics.

In addition, the shared virtual background further helped participants overcome avoidant screen gaze, in that outgroup distractions were reduced as no one in the group could see these beyond the shared background.

**S13:** *It [shared background] motivates me to present in the group [the] whole time without distracting* (Group 2, Transcription of Post-intervention, Student Comments, p. 1, lines 29–30).
**S29:** *A big focus for what we are doing here in the group work. It really helps us to concentrate what we are doing right now, at the moment. I'm not getting any distractions from our environment, or problems. So, as an example, if there's no background, I'm really sure that you also can see the cockroach that was attacking me. So, it distracts others too, so that it's a really good thing to have a background in future* (Group 5, Transcription of Post-intervention, Focus Group, p. 15, lines 540–545).

Furthermore, if a group member turned away from the screen and toward such a distraction (e.g., someone in their room speaking to them as occurred pre-intervention), that student's whole physical presence disappeared immediately and entirely from the group. This sensitivity of the shared background appeared to increase students' focus on their on-screen team members.

However, with the issues of network connection and supportiveness of devices (laptops and mobile phones), some students could not apply the common virtual background because they had to connect to the discussion through their phones. On such occasions, the other team members were seen (post but not pre-intervention) to make greater efforts to elicit input to discussion from students in this situation.

Overall, the above findings shed light on the advantages of the application of a shared virtual background in terms of avoiding background distractions as it facilitates students to focus on what they communicate as a group. Furthermore, this shared virtual background effect undermined the disadvantage of socio-economic differences that might otherwise be exposed to others in the group (e.g., if a student joins from their bedroom and another joins from a luxurious study room).

**S19:** …* like Palavi said, that's also true: we are focusing on others' faces, body with this common background and also … we all* [are] *in a [the] same level in a group or in a discussion, we are not like one or two persons in up or others in lower. Using this common background, we are in* [on] *the same level* (Group 3, Transcription of Post-intervention, Focus Group, p. 14, lines 492–495).
**S22:** *We are given the feeling that we are in the same environment and not like a distance like we are virtually connected, but that, like using the same background for all the people, it will give something like we're in same the same environment* (Group 4, Transcription of Post-intervention, Focus Group, p. 21, lines 731–734).

This too is compassionate “noticing” for the psychological safety of others, which appeared to mediate both social and learning experiences in the group, as follows.

##### 3.2.1.1. Learning experience across the group

If not looking at the screen, a speaker might fail to observe the non-verbal behaviors of students who may be signaling, even unconsciously, that they do not understand parts of the presentation, whether that is conceptual, or because of spoken English language errors or accent, or difficulties of English comprehension such as from the rapid speed of others' speech. Even a small frown or moving/turning of the head may signal to the speaker that they should repeat or/and rephrase a point. Observing these signals is useful, in particular, if listeners do not wish to verbally interrupt the presentation. Furthermore, if listeners do not understand and cannot signal potential difficulties non-verbally to a presenter who is not looking at them, a follow-up discussion might prove difficult. Not attending to non-verbal cues will therefore not only affect the listeners trying to communicate their difficulties in following what is said but the whole group's learning experience in terms of the quality of criticality of the discussion that follows because some members may lack the comprehension they needed to participate. Both groups experienced such a problem during the pre-intervention group meeting.

##### 3.2.1.2. Social experience across the group

i If the presenter does not sustain screen gaze with the listeners, this may cause the listening group members to dissociate from their compassionate role of supporting the current speaker. This is true for the screen gaze of all students in the group, particularly for the speaker. This may lead to there being no perceived necessity for listeners to sustain their own screen gaze because evidence of their attention to a speaker is not noticed by that speaker; then listeners may feel that their supportive behaviors are pointless. In the online group format, in particular, the listeners may then become more susceptible to distractions in their physical environment.
ii The speaker who does not sustain screen gaze with the listeners is most likely to also miss other highly communicative non-verbal signals of engagement from the listeners. Nodding and smiling are useful signals of understanding and/or encouragement to the speaker to continue. Turning/moving heads from side to side, frowning or expressions of puzzlement, or blank looks may be useful signals to the speaker that he/she is not communicating successfully at this moment, and should repeat, and/or rephrase, and/or slow down or simply stop and check understanding around the group.
iii If the listeners do not sustain screen gaze with the presenter/speaker and other group members, they fail to notice if the presenter/speaker needs any encouragement or support to continue or if any group member/s needs further explanations to understand. This failure to notice one another's behaviors might affect achieving group tasks.

### 3.3. Analyses of two questionnaires

Below are the findings from the two questionnaires that were analyzed using the Wilcoxon Signed-Rank Test.

#### 3.3.1. Statistical analysis of questionnaire 1—On group work behaviors

The findings show changes from pre-intervention negative group behaviors (itemized in the questionnaire) to more positive post-intervention behaviors, that were statistically significant as shown in Table 2. It could be seen that the changes related, respectively, to what the students observed of their own group work behaviors; what they observed of others' group work behaviors; what they reported of their confidence to engage in group discussion; and their views on the influence (if any) of group discussion behaviors on learning.

**Table 2:** Wilcoxon Signed-Rank Test results—Questionnaire 1: group work behaviors (the pre- vs. the post-intervention).

**Item no**.	**The negative group behaviors decreased from pre-intervention group meetings to post-intervention group meetings with statistical significance**	***p-*value**
	**Self-observation of group work behaviors**	
4.2	*Talking a lot so that others do not get many chances to speak*.	0.039
4.3	*Talking in silence when shyer members are getting ready to speak*.	0.028
4.7	*Talking over others*.	0.026
	**Observed behaviors of other group members**	
5.1	*Talking a lot so that others do not get many chances to speak*.	0.003
5.2	*Talking in silence when shyer members are getting ready to speak*.	0.017
5.4	*Using difficult language terms or expressions without explaining so that other people in the group may not understand*.	0.010
5.5	*Not listening carefully to other peoples' ideas*.	0.009
5.6	*Not helping other people when they are getting into difficulty while they are speaking*.	0.003
5.7	*Talking over others*.	0.003
5.8	*Not inviting others to speak*.	0.010
5.10	*Speaking very little or not at all in the group*.	0.011
5.11	*Not even reading a little bit to bring something to the discussion*.	0.020
5.12	*Letting other people talk and talk without interrupting them*.	0.030
	**Confidence in drawing others into the discussion**	
6.2	*How confident are you to draw others into group discussion?*	0.011

These results may suggest an increase in students' “noticing” their own less helpful behaviors in group discussions.

#### 3.3.2. Statistical analysis of questionnaire on compassionate engagement and action scale

The Compassionate Engagement and Action Scale developed by the Compassionate Mind Foundation identifies three aspects of compassion. They are self-compassion [contrasting strongly with the destructive competitive individualistic elements of self-esteem (Neff et al., 2007; Kingston, 2008)]; sensitivity to (recognition of) compassion received from others; and compassion for others. All three types of compassion are known to mediate each other (Compassionate Mind Foundation).

A comparison of data collected from the above questionnaire, before and after the CSCC intervention session, was made through the Wilcoxon Signed-Rank Test. Results indicated a statistically significant difference of *p* = 0.5 in students' responses (between pre- and post-CSCC) to the Compassionate Engagement and Action Scale in all three types of compassion, as in Table 3.

**Table 3:** Wilcoxon Signed-Rank Test results—Compassionate engagement and action scale (the pre vs. the post-intervention).

**Item no**.	**Items of the CEAS for which there was a statistically significant positive change in responses after the post-intervention meeting**	***p*-value**
	**Self-compassion**	
1.	*I am motivated to engage and work with my distress when it arises*.	0.007
10.	*I think about and come up with helpful ways to cope with my distress*.	0.007
	**Compassion for others**.	
14.	*I am motivated to engage and work with other peoples' distress when it arises*.	0.038
	**Compassion from others**	
36.	*Others think about and come up with helpful ways for me to cope with my distress*.	0.050

Taken together, the two questionnaires offered further opportunities to better identify and explore changes in the respondents' experiences of self and others that might be attributable to the CSCC intervention session for online group work.

The data sets from both indicated enhancements to group members' noticing of their own and others' team meeting behaviors. “Noticing” problematic behaviors is an important component of the definition of compassion on which the intervention pedagogy is based.

Overall, the results of this study are that before the respondents were introduced to compassionate team communication strategies, there were three particular barriers to their communicative ease with each other in their online meetings. These three appeared the most effective at dissociating students from each other in their meetings. There was, first, reluctance by some participants to switch on their cameras even if their internet connections were not a problem, and this is widely reported in the literature. The second was external distractions, including communications with non-group members by listeners. The third was those article presenters, who sometimes offered very limited screen gaze attention to the group or its responses to what was being presented because of the speaker's over-reliance on reading, head down, from notes.

The study has identified that after an intervention that introduced the students to using the science of compassion with communicative strategies that they could use for themselves online, the two explicit key components of compassion (*noticing* distress or disadvantage and taking *wise* action to reduce or prevent them) were seen consistently practiced across the group, in contrast to what was seen in the earlier meetings. There was a shift in the nature of many of their verbal and non-verbal communications during their substantially increased (post-intervention) screen gaze attention to their groups. To be clear, the findings informed the change in students' previous negative group behaviors (inequality of sharing speaking time/dominating, interruptions, competitive individualism, and non-contributing) with more inclusive and collaborative interactions. These same negative group behaviors have also been identified across disciplines in the HE classroom seminar/tutorial (Gilbert, 2016; Harvey et al., 2020).

Interestingly, the findings suggest that training the students in CSCC motivated them to use practical compassionate communication to manage their group/teamwork interactions irrespective of their ethnic, religious, or mother tongue differences. This may be the result of compassion being a universally valued concept cross-culturally (Schwartz and Bardi, 2001; Immordino-Yang and Damasio, 2007; Goetz et al., 2010; Davidson and Harrington, 2012; Van der Cingel, 2014). Similar findings in the research on compassion can be identified in previous literature where Neff et al. (2007), for example, found extensive benefits to the student of having a more interdependent self-concept.

Hence, the above all suggests changes in the neurobiological affiliation processing of the individuals in this study where it appears that the stimulation of the capacity for self-compassion helped to downregulate the brain's social threat alert system (Compassionate Mind Foundation). This happens through the release of oxytocin in the brain (Depue and Morrone-Strupinsky, 2005; Uvnäs-Moberg et al., 2015) enabling self-soothing. Colonnello et al. (2017) explain that the release of oxytocin plays a major role in supporting individuals in teams to synchronize with each other communicatively, including in anticipatory ways that aid group communications. This is relevant when students can see one another and employ compassionate non-verbal cues to promote equal participation in class discussions (e.g., through screen gaze, nodding, listening attentively, or showing understanding, disagreement, encouragement, or confusion as people speak). According to Jensen et al. (2013), the synchronizing of group members helping out a single person may be connected to the part that oxytocin plays in the brain's reward system.

## 4. Discussion

One of the main difficulties of online group work appeared to be constraints on the way eye contact can be used for compassionate communications practice in the classroom, where, whenever they speak, students are encouraged to “sweep” the group with eye contact as if it were a single organism (Gilbert, 2018). In line with evolutionary findings by Dal Monte et al. (2022), this has been shown not just to help the speaker read the group's understanding, but also to signal each listener that they are being spoken to individually in ways that support psychological safety. This is key in helping equalize participation around the group (Gilbert et al., 2018; Gilbert and Bryan, 2019). Screen gaze online cannot be used to observably “sweep” the group inclusively like this. Nevertheless, during the post-intervention group meetings, screen gaze was sustained much more across all groups than in the pre-intervention group meetings. Something that may have aided this is that in online, it is possible to read all faces in one single screen-sized space.

Moreover, it is important to remember that what can be seen online (faces, provided the camera is on) was evolutionarily designed to be read closely. This requires the first component of compassion and its focus on noticing. Recent research by Spikins (2015) and Godinho et al. (2018) on the evolution of human facial expressions suggests that modern humans have developed quizzical eyebrows (as *Homo sapiens* lost the strong, thick bony brow ridges of their ancient ancestors) as a result of human evolution where effective social communication in hunter-gatherer teams became important. From this, Spikins (2015) and also Godinho et al. (2018) conclude that the evolution of smaller, flatter faces may have facilitated the social power of the eyebrow, allowing humans to communicate at a distance in more complex and nuanced ways as muscles in the face developed to move the eyebrows up and down expressively for more subtle communications.

Pertinently, Dal Monte et al. (2022) have found that eye contact between people has sophisticated neurological correlates that have evolved in our social brains for deriving significance from other people's gazes. They have identified that extracting meaning from social gaze contact involves neurons in four brain regions and have highlighted the significant influence of social gaze interaction in shaping interpersonal communications. This may explain why even online the CSCC appeared to motivate students to take care to assess responses and overall reactions of the whole group to presentations or discussions. In other words, the nature of gaze reading is changed online, but it is still crucial, and therefore also the use of cameras. Note that this is a matter of non-verbal social connectivity.

The verbal evidence for enhanced inter students' support of each other similarly aligned with the principles of non-verbal compassionate communication, as mentioned above. The study's findings are therefore in line with Vertegaal and Ding (2002), Vertegaal et al. (2002) research. Their eye-tracking study explored the role of eye gaze in group work via video conferencing. They found that when all team members believed that the speaker was looking only at them, the participation of the group members equalized and the quality of problem-solving and decision-making was enhanced (Vertegaal et al., 2002, 2003).

It is important to enact the first component of compassion for the group work/teamwork context (noticing) to fulfill the second component (taking wise actions to reduce or prevent the distress or disadvantaging of self and others). Understanding this phenomenon and the application of the CSCC practically during the post-intervention task-focused group work meetings assisted students' recognition of the advantages of switching their cameras on during their group/team meetings online. This was especially enhanced by their realization that they could offer a wide range of support to their peers by implementing what they had learned about CSCC. Hence, this approach appears to help address the multi-factorial issue of delayed or abandoned development of social relationships that could be remedied through even non-verbal exchanges in online group meetings (Butz et al., 2015; Bedenlier et al., 2020; Khalil et al., 2020). Furthermore, the current findings are important for addressing negative emotions including feelings of isolation and/or helplessness by students having had to shift to online platforms (Bedenlier et al., 2020).

Moreover, as this study has found, online meetings can offer other ways to support inclusivity and psychological safety that also help equalize participation and students' use of CSCC in online group meetings appeared to enhance group cohesion, inclusivity, and the notion of equal agency. In addition, group interactivity was statistically found to be less vulnerable to external environment disruptions.

This study on compassion as an intention (not an emotion; Compassionate Mind Foundation) suggests new avenues to enhance the productivity and inclusivity of online group work/teamwork meetings. For example, in a group of four members with cameras on, each group member can read the expressions, all on one screen, of all three other members of the group, at the same time. Compared to when a group sits around the table, this is a change of spatial dimensions for “reading” faces and their non-verbal cues and signals (e.g., confusion, approval, disagreement, and encouragement) during the meeting.

This alone may be worthy of further research in terms of how the observing social brain adapts under compassionate conditions, where oxytocin may help sync the group (Colonnello et al., 2017). This is important because of research such as that of Greenfield (2010) on identifying how the current, widespread requirement for daily digital multi-focusing is changing the architecture of children's brains in digital societies. She asserts, “if you only focused on the behavior of one player (in a game of football, for example) you couldn't extrapolate the nature and context of the game.” Similarly, in their group meetings, if students focus on the speaker only (which often happens in non-CSCC-informed offline meetings), they may not also pay close attention to the immediate facial responses of the rest of the group members. However, this advantage of reading faces (in online group meetings) is only possible when attendees have their cameras switched on. Dal Monte et al. (2022) have found that eye contact between people has sophisticated neurological correlates in the human brain that have evolved in our social brains for deriving significance from other people's gazes. They have identified that extracting meaning from social gaze contact involves neurons in four brain regions and highlighted the significant influence of social gaze interaction in shaping interpersonal communications. Thus, in online meetings to express one's own non-verbal communication (especially facial expressions) while monitoring the responses and interactions of other group members, online camera use is important.

The study strongly suggested that monopolizing behavior indicates that there has been an initial activation of the threat system, remembering that the monopoliser in team meetings may often be the most anxious person in the team (Yalom and Leszsz, 2005). This is entirely plausible given the highly individualistically competitive nature of HE (Greenfield, 2010). The monopolizing behavior itself can be understood to be an outcome of the brain's drive system for successful performativity.

Finally, the study's findings demonstrated the successful adaptation of the CSCC to the online group meeting context. The findings also support the notion of Self Organized Learning Environments (SOLE) introduced by Mitra and Dangwal (2017) and Mitra (2018). In the context of this study, “self-organized” is inferred from the choice by students of the journal articles to present and discuss without a tutor taking part. The whole point of the discussion was to develop critical perspectives taken through the social interaction (in this case based on an empirical understanding of compassion) considered by the constructivists as necessary for student learning.

## 5. Conclusion

Limitations of this study are that it was a relatively small scale and the results of this study might not be replicated with a sample of students not in the same socio-political environment as Sri Lankan students. Moreover, follow-up of the students involved has not yet been completed to identify whether the skills they learned have helped them since. The follow-up is planned but may be complicated by new circumstances in Sri Lanka around its recent, well-documented economic crisis, during which many of its HE teaching staff have left the country (Agalakada, 2023) and this will be having a material effect on student wellbeing and success. But the study findings align with those of other studies of the motivational nature of cognitive compassion in classroom group/teamwork; it invites further research given the clear relevance of an empirically understood concept of compassion—or its absence—to team meeting processes.

The findings indicate the value of raising students' awareness of the cognitive skills of compassionate communication that they can use in their online group meetings. The results of the study have identified that after this intervention, all students in the sample were motivated to turn on their cameras and to sustain their own observable screen gaze attention to their groups, in contrast, to do what they did pre-intervention. This, in turn, did appear to develop their own group observation skill, including interpreting other group members' verbal and non-verbal meta-language communications across the group. Evidence of students' engagement with each other's presentations substantially increased with their application of CSCC and that engagement was seen in the evidence of increased understanding of the content of the presentations (This also evidenced a useful and important integration of their social and learning experiences in their task-focused meetings, post-intervention). Overall, the findings suggest that training the students in CSCC motivates them to use practical compassionate communication to manage their group/teamwork interactions irrespective of their ethnic, religious, mother tongue, or gender differences. Furthermore, the shared virtual backgrounds that are a benefit of *online* meetings created a unity of circumstance for each student and it is notable that they seemed pleased without exception to be working outside their COVID-19-mediated physically confined environments. This was an example of how the adaptation of the CSCC was much helped through a partnership with the students. We furthermore suggest that the shared background likely had some effect on the willingness of one or more students in the sample to switch on their cameras not least in relation to different socio-economic backgrounds that non-use of this background-sharing exposed.

This is important because if students' cameras are switched off, tutors might not be able to identify who is speaking, or prompting, who may be supporting the speaker unseen in their physical location, or whether a group member is script-reading when they speak to the group. Hence, the findings of this study contribute to addressing the significant dearth of existing research on the current use of video-conferencing in higher education (Al-Samarraie, 2019, p. 122) to effectively address a known, fundamental challenge that presents in similar forms: disconnection among and between students (Wang et al., 2018; Bauer et al., 2020; Stanford University, 2020), feeling isolation/loneliness and the resulting negative psychological consequences, entrenching disconnection among and between students through online delivery of higher education (Schwenck and Pryor, 2021), lack of consistently satisfying experience for students in online educational settings due to isolation and limited interactions because of their reluctance to switch their cameras on Young and Bruce (2020). Kim et al. (2011) and Kim (2013) indicate lower levels of interactivity and less in-depth discussions. Alongside this, Author:inner group AEDiL (2021) reports on instructors feeling insecure, helpless, and frustrated as a result of students' not switching on their cameras.

With the introduction by the UK government of new restrictions on the movement of international students into UK HE, it is recommended that further studies on the nature of cognitive compassion in teamwork be carried out in other countries to best support students where they are. This study may be helpful in that regard as a methodological model, bearing in mind that not all the methods used here (to support triangulation in this study) need to be used. We suggest that for such further studies, the Microsoft Excel analysis, for example, might not be essential.

Hence, in terms of achieving better outcomes by addressing the existing issues in online group work meetings, CSCC could be a way forward. This can be not just for teaching and learning.

## Data availability statement

The original contributions presented in the study are included in the article/Supplementary material, further inquiries can be directed to the corresponding author/s.

## Ethics statement

The study has been reviewed by the Ethics Committee of the University of Hertfordshire (protocol code: cHUM/PGT/UH/04345, (1), (2) issued on 3 October 2019, 28 February 2020, and 20 April 2022 respectively). The patients/participants provided their written informed consent to participate in this study.

## Author contributions

JJ: conceptualization, methodology, formal analysis, investigation, resources, data curation, writing—original draft preparation, writing—review and editing, visualization, project administration, and funding acquisition. TG, SK, and LM: supervision. All authors contributed to the article and approved the submitted version.

## Footnotes

^1^A group meeting consisted of each group member presenting a self-chosen journal article followed up immediately after by the whole group's discussion of that article as presented.

^2^Mainly focus groups were conducted with each group before and after the CSCC training. However, interviews were conducted with those who had some technical difficulties or other commitments where they could not be present for the whole of the focus group meeting.

^3^A pilot study, referred to in the study (Jayasundara et al., 2022), as Cycle 1 was conducted prior to this study (Cycles 2 and 3) as part of an, overall, action research project. The pilot/Cycle 1 was carried out among two groups of international/Sri Lankan STEM students (*n* = 8) studying in five UK universities in preparation for the study reported here.

## Supplementary material

Supplementary Table 1 Compassionate engagement and action scale.

Supplementary Table 2 Questionnaire on group work behaviors.
