# Supplementary material for: Why should I switch on my camera? Developing the cognitive skills of compassionate communications for online group/teamwork management
Source: Front Psychol. 2023 Aug 4;14:1113098. doi: 10.3389/fpsyg.2023.1113098 (PMC10436525; doi:10.3389/fpsyg.2023.1113098)
Supplement: Supplementary Table 1 — Compassionate engagement and action scale. [file Table_1.docx]

**Supplementary Material 1**

## The Compassionate Engagement and Action Scale

**The Compassionate Engagement and Action Scales**

This short, anonymous survey asks questions about Self-compassion, (Section 1), Compassion to others (Section 2) and Sensitivity to compassion from others (Section 3). Could you kindly respond to the questions below? This will take approximately 10 minutes. The results from the survey will help us identify each individual participant’s engagement with, and then action, if any, in relation to: Self-compassion; sensitivity/receptiveness to compassion from others, and compassion for others. The work is approved by the University of Hertfordshire Social Sciences, Arts and Humanities Ethics Committee with Delegated Authority, UH protocol No. cHUM/PGT/UH/04345.

You are free to withdraw at any stage, just stop answering the questionnaire or leave this page.

What will happen to the data collected within this study?

• The data collected will be stored electronically, in a password-protected environment, for four years, after which time it will be destroyed under secure conditions.

• The data will be analysed, and the results will be used in publications and presentations. The analysis will contribute to the primary researcher's PhD project.

In the box below, please enter the code given to you by the researcher.Top of Form

## Self-Compassion

When things go wrong for us and we become distressed by setbacks, failures, disappointments or losses, we may cope with these in different ways. We are interested in the degree to which people can be compassionate with themselves. We define compassion as “a sensitivity to suffering in self and others with a commitment to try to alleviate and prevent it".

This means there are two aspects to compassion. The first is the ability to be motivated to engage with things/feelings that are difficult as opposed to trying to avoid or supress them. The second aspect of compassion is the ability to focus on what is helpful to us. Just like doctors with their patients. In other words, the first aspect of compassion is to be motivated and able to pay attention to the pain and (learn how to) make sense of it. The second is to be able to take the action that will be helpful. Below is a series of questions that ask you about these two aspects of compassion. Therefore, read each statement carefully and think about how it applies to you if you become distressed.

Section 1 – These are questions that ask you about how motivated you are, and able to engage with distress when you experience it. So:  
 When I’m distressed or upset by things…

1. I am motivated to engage and work with my distress when it arises.Top of Form

|  | 1 | 2 | 3 | 4 | 5 | 6 | 7 | 8 | 9 | 10 |  |
| --- | --- | --- | --- | --- | --- | --- | --- | --- | --- | --- | --- |
| Never |  |  |  |  |  |  |  |  |  |  | Always |

1. I notice, and am sensitive to, my distressed feelings when they arise in me.

|  | 1 | 2 | 3 | 4 | 5 | 6 | 7 | 8 | 9 | 10 |  |
| --- | --- | --- | --- | --- | --- | --- | --- | --- | --- | --- | --- |
| Never |  |  |  |  |  |  |  |  |  |  | Always |

1. I avoid thinking about my distress and try to distract myself and put it out of my mind.

|  | 1 | 2 | 3 | 4 | 5 | 6 | 7 | 8 | 9 | 10 |  |
| --- | --- | --- | --- | --- | --- | --- | --- | --- | --- | --- | --- |
| Never |  |  |  |  |  |  |  |  |  |  | Always |

1. I am emotionally moved by my distressed feelings or situations.

|  | 1 | 2 | 3 | 4 | 5 | 6 | 7 | 8 | 9 | 10 |  |
| --- | --- | --- | --- | --- | --- | --- | --- | --- | --- | --- | --- |
| Never |  |  |  |  |  |  |  |  |  |  | Always |

1. I tolerate the various feelings that are part of my distress.

|  | 1 | 2 | 3 | 4 | 5 | 6 | 7 | 8 | 9 | 10 |  |
| --- | --- | --- | --- | --- | --- | --- | --- | --- | --- | --- | --- |
| Never |  |  |  |  |  |  |  |  |  |  | Always |

1. I reflect on and make sense of my feelings of distress.

|  | 1 | 2 | 3 | 4 | 5 | 6 | 7 | 8 | 9 | 10 |  |
| --- | --- | --- | --- | --- | --- | --- | --- | --- | --- | --- | --- |
| Never |  |  |  |  |  |  |  |  |  |  | Always |

1. I do not tolerate being distressed.

|  | 1 | 2 | 3 | 4 | 5 | 6 | 7 | 8 | 9 | 10 |  |
| --- | --- | --- | --- | --- | --- | --- | --- | --- | --- | --- | --- |
| Never |  |  |  |  |  |  |  |  |  |  | Always |

1. I am accepting, non-critical and non-judgemental of my feelings of distress.

|  | 1 | 2 | 3 | 4 | 5 | 6 | 7 | 8 | 9 | 10 |  |
| --- | --- | --- | --- | --- | --- | --- | --- | --- | --- | --- | --- |
| Never |  |  |  |  |  |  |  |  |  |  | Always |

Section 2 – These questions relate to how you actively cope in compassionate ways with emotions, thoughts and situations that distress you. So:

When I’m distressed or upset by things…

1. I direct my attention to what is likely to be helpful to me.

|  | 1 | 2 | 3 | 4 | 5 | 6 | 7 | 8 | 9 | 10 |  |
| --- | --- | --- | --- | --- | --- | --- | --- | --- | --- | --- | --- |
| Never |  |  |  |  |  |  |  |  |  |  | Always |

1. I think about and come up with helpful ways to cope with my distress.

|  | 1 | 2 | 3 | 4 | 5 | 6 | 7 | 8 | 9 | 10 |  |
| --- | --- | --- | --- | --- | --- | --- | --- | --- | --- | --- | --- |
| Never |  |  |  |  |  |  |  |  |  |  | Always |

1. I don’t know how to help myself.

|  | 1 | 2 | 3 | 4 | 5 | 6 | 7 | 8 | 9 | 10 |  |
| --- | --- | --- | --- | --- | --- | --- | --- | --- | --- | --- | --- |
| Never |  |  |  |  |  |  |  |  |  |  | Always |

1. I take the actions and do the things that will be helpful to me.

|  | 1 | 2 | 3 | 4 | 5 | 6 | 7 | 8 | 9 | 10 |  |
| --- | --- | --- | --- | --- | --- | --- | --- | --- | --- | --- | --- |
| Never |  |  |  |  |  |  |  |  |  |  | Always |

1. I create inner feelings of support, helpfulness and encouragement.

|  | 1 | 2 | 3 | 4 | 5 | 6 | 7 | 8 | 9 | 10 |  |
| --- | --- | --- | --- | --- | --- | --- | --- | --- | --- | --- | --- |
| Never |  |  |  |  |  |  |  |  |  |  | Always |

## Compassion to Others

When things go wrong for us and we become distressed by setbacks, failures, disappointments or losses, we may cope with these in different ways. We are interested in the degree to which people can be compassionate to others. We define compassion as “a sensitivity to suffering in self and others with a commitment to try to alleviate and prevent it".

This means there are two aspects to compassion. The first is the ability to be motivated to engage with things/feelings that are difficult as opposed to trying to avoid or supress them. The second aspect of compassion is the ability to focus on what is helpful to us. Just like doctors with their patients. In other words, the first aspect of compassion is to be motivated and able to pay attention to the pain and (learn how to) make sense of it. The second is to be able to take the action that will be helpful. Below is a series of questions that ask you about these two aspects of compassion. Therefore, read each statement carefully and think about how it applies to you when people in your life become distressed.

Section 1 – These are questions that ask you about how motivated you are, and able to engage with distress when you experience it. So:

When I’m distressed or upset by things…

1. I am motivated to engage and work with other peoples’ distress when it arises.

|  | 1 | 2 | 3 | 4 | 5 | 6 | 7 | 8 | 9 | 10 |  |
| --- | --- | --- | --- | --- | --- | --- | --- | --- | --- | --- | --- |
| Never |  |  |  |  |  |  |  |  |  |  | Always |

1. I notice and am sensitive to distress in others when it arises.

|  | 1 | 2 | 3 | 4 | 5 | 6 | 7 | 8 | 9 | 10 |  |
| --- | --- | --- | --- | --- | --- | --- | --- | --- | --- | --- | --- |
| Never |  |  |  |  |  |  |  |  |  |  | Always |

1. I avoid thinking about other peoples’ distress, try to distract myself and put it out of my mind.

|  | 1 | 2 | 3 | 4 | 5 | 6 | 7 | 8 | 9 | 10 |  |
| --- | --- | --- | --- | --- | --- | --- | --- | --- | --- | --- | --- |
| Never |  |  |  |  |  |  |  |  |  |  | Always |

1. I am emotionally moved by expressions of distress in others.

|  | 1 | 2 | 3 | 4 | 5 | 6 | 7 | 8 | 9 | 10 |  |
| --- | --- | --- | --- | --- | --- | --- | --- | --- | --- | --- | --- |
| Never |  |  |  |  |  |  |  |  |  |  | Always |

1. I tolerate the various feelings that are part of other people’s distress.

|  | 1 | 2 | 3 | 4 | 5 | 6 | 7 | 8 | 9 | 10 |  |
| --- | --- | --- | --- | --- | --- | --- | --- | --- | --- | --- | --- |
| Never |  |  |  |  |  |  |  |  |  |  | Always |

1. I reflect on and make sense of other people’s distress.

|  | 1 | 2 | 3 | 4 | 5 | 6 | 7 | 8 | 9 | 10 |  |
| --- | --- | --- | --- | --- | --- | --- | --- | --- | --- | --- | --- |
| Never |  |  |  |  |  |  |  |  |  |  | Always |

1. I do not tolerate other peoples’ distress.

|  | 1 | 2 | 3 | 4 | 5 | 6 | 7 | 8 | 9 | 10 |  |
| --- | --- | --- | --- | --- | --- | --- | --- | --- | --- | --- | --- |
| Never |  |  |  |  |  |  |  |  |  |  | Always |

1. I am accepting, non-critical and non-judgemental of other people’s distress.

|  | 1 | 2 | 3 | 4 | 5 | 6 | 7 | 8 | 9 | 10 |  |
| --- | --- | --- | --- | --- | --- | --- | --- | --- | --- | --- | --- |
| Never |  |  |  |  |  |  |  |  |  |  | Always |

Section 2 – These questions relate to how you actively respond in compassionate ways when other people are distressed. So:

When others are distressed or upset by things…

1. I direct attention to what is likely to be helpful to others.

|  | 1 | 2 | 3 | 4 | 5 | 6 | 7 | 8 | 9 | 10 |  |
| --- | --- | --- | --- | --- | --- | --- | --- | --- | --- | --- | --- |
| Never |  |  |  |  |  |  |  |  |  |  | Always |

1. I think about and come up with helpful ways for them to cope with their distress.

|  | 1 | 2 | 3 | 4 | 5 | 6 | 7 | 8 | 9 | 10 |  |
| --- | --- | --- | --- | --- | --- | --- | --- | --- | --- | --- | --- |
| Never |  |  |  |  |  |  |  |  |  |  | Always |

1. I take the actions and do the things that will be helpful to others.

|  | 1 | 2 | 3 | 4 | 5 | 6 | 7 | 8 | 9 | 10 |  |
| --- | --- | --- | --- | --- | --- | --- | --- | --- | --- | --- | --- |
| Never |  |  |  |  |  |  |  |  |  |  | Always |

1. I don’t know how to help other people when they are distressed.

|  | 1 | 2 | 3 | 4 | 5 | 6 | 7 | 8 | 9 | 10 |  |
| --- | --- | --- | --- | --- | --- | --- | --- | --- | --- | --- | --- |
| Never |  |  |  |  |  |  |  |  |  |  | Always |

1. I express feelings of support, helpfulness and encouragement to others.

|  | 1 | 2 | 3 | 4 | 5 | 6 | 7 | 8 | 9 | 10 |  |
| --- | --- | --- | --- | --- | --- | --- | --- | --- | --- | --- | --- |
| Never |  |  |  |  |  |  |  |  |  |  | Always |

## Compassion from Others

When things go wrong for us and we become distressed by setbacks, failures, disappointments or losses, others may cope with our distress in different ways. We are interested in the degree to which you feel that important people in your life can be compassionate to your distress. We define compassion as “a sensitivity to suffering in self and others with a commitment to try to alleviate and prevent it".

This means there are two aspects to compassion. The first is the ability to be motivated to engage with things/feelings that are difficult as opposed to trying to avoid or supress them. The second aspect of compassion is the ability to focus on what is helpful to us or others. Just like doctors with their patients. In other words, the first aspect of compassion is to be motivated and able to pay attention to the pain and (learn how to) make sense of it. The second is to be able to take the action that will be helpful. Below is a series of questions that ask you about these two aspects of compassion. Therefore, read each statement carefully and think about how it applies to the important people in your life when you become distressed.

Section 1 – These are questions that ask you about how motivated you think others are, and how much they engage with your distress when you experience it. So:

When I’m distressed or upset by things…

1. Other people are actively motivated to engage and work with my distress when it arises.

|  | 1 | 2 | 3 | 4 | 5 | 6 | 7 | 8 | 9 | 10 |  |
| --- | --- | --- | --- | --- | --- | --- | --- | --- | --- | --- | --- |
| Never |  |  |  |  |  |  |  |  |  |  | Always |

1. Others notice and are sensitive to my distressed feelings when they arise in me.

|  | 1 | 2 | 3 | 4 | 5 | 6 | 7 | 8 | 9 | 10 |  |
| --- | --- | --- | --- | --- | --- | --- | --- | --- | --- | --- | --- |
| Never |  |  |  |  |  |  |  |  |  |  | Always |

1. Others avoid thinking about my distress, try to distract themselves and put it out of their mind.

|  | 1 | 2 | 3 | 4 | 5 | 6 | 7 | 8 | 9 | 10 |  |
| --- | --- | --- | --- | --- | --- | --- | --- | --- | --- | --- | --- |
| Never |  |  |  |  |  |  |  |  |  |  | Always |

1. Others are emotionally moved by my distressed feelings.

|  | 1 | 2 | 3 | 4 | 5 | 6 | 7 | 8 | 9 | 10 |  |
| --- | --- | --- | --- | --- | --- | --- | --- | --- | --- | --- | --- |
| Never |  |  |  |  |  |  |  |  |  |  | Always |

1. Others tolerate my various feelings that are part of my distress.

|  | 1 | 2 | 3 | 4 | 5 | 6 | 7 | 8 | 9 | 10 |  |
| --- | --- | --- | --- | --- | --- | --- | --- | --- | --- | --- | --- |
| Never |  |  |  |  |  |  |  |  |  |  | Always |

1. Others reflect on and make sense of my feelings of distress.

|  | 1 | 2 | 3 | 4 | 5 | 6 | 7 | 8 | 9 | 10 |  |
| --- | --- | --- | --- | --- | --- | --- | --- | --- | --- | --- | --- |
| Never |  |  |  |  |  |  |  |  |  |  | Always |

1. Others do not tolerate my distress.

|  | 1 | 2 | 3 | 4 | 5 | 6 | 7 | 8 | 9 | 10 |  |
| --- | --- | --- | --- | --- | --- | --- | --- | --- | --- | --- | --- |
| Never |  |  |  |  |  |  |  |  |  |  | Always |

1. Others are accepting, non-critical and non-judgemental of my feelings of distress.

|  | 1 | 2 | 3 | 4 | 5 | 6 | 7 | 8 | 9 | 10 |  |
| --- | --- | --- | --- | --- | --- | --- | --- | --- | --- | --- | --- |
| Never |  |  |  |  |  |  |  |  |  |  | Always |

Section 2 – These questions relate to how others actively cope in compassionate ways with emotions and situations that distress you. So:

When I’m distressed or upset by things…

1. Others direct their attention to what is likely to be helpful to me.

|  | 1 | 2 | 3 | 4 | 5 | 6 | 7 | 8 | 9 | 10 |  |
| --- | --- | --- | --- | --- | --- | --- | --- | --- | --- | --- | --- |
| Never |  |  |  |  |  |  |  |  |  |  | Always |

1. Others think about and come up with helpful ways for me to cope with my distress.

|  | 1 | 2 | 3 | 4 | 5 | 6 | 7 | 8 | 9 | 10 |  |
| --- | --- | --- | --- | --- | --- | --- | --- | --- | --- | --- | --- |
| Never |  |  |  |  |  |  |  |  |  |  | Always |

1. Others don’t know how to help me when I am distressed.

|  | 1 | 2 | 3 | 4 | 5 | 6 | 7 | 8 | 9 | 10 |  |
| --- | --- | --- | --- | --- | --- | --- | --- | --- | --- | --- | --- |
| Never |  |  |  |  |  |  |  |  |  |  | Always |

1. Others take the actions and do the things that will be helpful to me.

|  | 1 | 2 | 3 | 4 | 5 | 6 | 7 | 8 | 9 | 10 |  |
| --- | --- | --- | --- | --- | --- | --- | --- | --- | --- | --- | --- |
| Never |  |  |  |  |  |  |  |  |  |  | Always |

1. Others treat me with feelings of support, helpfulness and encouragement.Top of Form

|  | 1 | 2 | 3 | 4 | 5 | 6 | 7 | 8 | 9 | 10 |  |
| --- | --- | --- | --- | --- | --- | --- | --- | --- | --- | --- | --- |
| Never |  |  |  |  |  |  |  |  |  |  | Always |

Thank you very much for your participation and time spent on this survey.
