# Supplementary material for: Why should I switch on my camera? Developing the cognitive skills of compassionate communications for online group/teamwork management
Source: Front Psychol. 2023 Aug 4;14:1113098. doi: 10.3389/fpsyg.2023.1113098 (PMC10436525; doi:10.3389/fpsyg.2023.1113098)
Supplement: Supplementary Table 2 — Questionnaire on group work behaviors. [file Table_2.docx]

**Supplementary Material 2**

## Questionnaire on Group Work Behaviours

**Questionnaire on Group Work Behaviours**

This short, anonymous survey asks questions about group work.  Could you kindly spend approximately 05 minutes sharing your opinions, please?  The results from the survey (pre and post) will help us identify the impact, if any, of the evidenced based compassionate pedagogy to support students’ communicative ease with others in groupwork. The work is approved by the University of Hertfordshire Social Sciences, Arts and Humanities Ethics Committee with Delegated Authority, UH protocol No. cHUM/PGT/UH/04345.

You are free to withdraw at any stage, just stop answering the questionnaire or leave this page.

What will happen to the data collected within this study?

• The data collected will be stored electronically, in a password-protected environment, for four years, after which time it will be destroyed under secure conditions.

• The data will be analysed and the results will be used in publications and presentations. The analysis will contribute to the primary researcher's PhD project.

1. In the box below, please enter the code given to you by the researcher.

## Demographic Information

## Which age group do you belong to?

## 18 - 25

## 26 - 35

## 36 - 45

## 46 - 55

## 56 and above

## Prefer not to say

## What is your gender?

## Male

## Female

## Other

## Prefer not to say

## In your view, which of the following best describes your level of English?

## Expert user - accurate, appropriate, fluent with full understanding

## Very good user - rare errors, use complex language well

## Good user - only occasional errors, use complex language quite well in most situations

## Competent user - some errors, use some complex language in familiar situations

## Modest user - frequent errors, have difficulties with complex language

1. Please tick any of the following behaviours that you have demonstrated (your own behaviour) in your group discussions.

|  | Negative Group Behaviours | Always (1) | Quiet often (2) | Sometimes (3) | Not very often (4) | Never (5) |
| --- | --- | --- | --- | --- | --- | --- |
| 4.1 | Talking a lot so that others do not get many chances to speak. |  |  |  |  |  |
| 4.2 | Talking in silences when shyer members are getting ready to speak. |  |  |  |  |  |
| 4.3 | Not looking at all the other people in the group. |  |  |  |  |  |
| 4.4 | Using difficult language terms or expressions without explaining so that other people in the group may not understand. |  |  |  |  |  |
| 4.5 | Not listening carefully to other peoples' ideas. |  |  |  |  |  |
| 4.6 | Not helping other people when they are getting into difficulty while they are speaking. |  |  |  |  |  |
| 4.7 | Talking over others. |  |  |  |  |  |
| 4.8 | Not inviting others to speak. |  |  |  |  |  |
| 4.9 | Not thanking others for their contribution. |  |  |  |  |  |
| 4.10 | Speaking very little or not at all in the group. |  |  |  |  |  |
| 4.11 | Not even reading a little bit in order to bring something to the discussion. |  |  |  |  |  |
| 4.12 | Letting other people talk and talk without interrupting them. |  |  |  |  |  |
| 4.12 | Letting other people talk and talk without interrupting them. |  |  |  |  |  |
| 4.13 | Allowing others to speak too fast for everyone to understand them. |  |  |  |  |  |
| 4.14 | Not asking for more explanations when understanding becomes too difficult. |  |  |  |  |  |
| 4.15 | Other: |  |  |  |  |  |

4a. If you selected the item 15 'Other', please include your observations here.

1. Please tick any of the following behaviours that others have demonstrated (you have observed in others) in your group discussions.

|  | Negative Group Behaviours | Always (1) | Quiet often (2) | Sometimes (3) | Not very often (4) | Never (5) |
| --- | --- | --- | --- | --- | --- | --- |
| 5.1 | Talking a lot so that others do not get many chances to speak. |  |  |  |  |  |
| 5.2 | Talking in silences when other group members are talking. |  |  |  |  |  |
| 5.3 | Not looking at all the other people in the group. |  |  |  |  |  |
| 5.4 | Using difficult language terms or expressions without explaining so that other people in the group may not understand. |  |  |  |  |  |
| 5.5 | Not listening carefully to other peoples' ideas. |  |  |  |  |  |
| 5.6 | Not helping other people when they are getting into difficulty while they are speaking. |  |  |  |  |  |
| 5.7 | Talking over others. |  |  |  |  |  |
| 5.8 | Not inviting others to speak. |  |  |  |  |  |
| 5.9 | Not thanking others for their contribution. |  |  |  |  |  |
| 5.10 | Speaking very little or not at all in the group. |  |  |  |  |  |
| 5.11 | Not even reading a little bit in order to bring something to the discussion. |  |  |  |  |  |
| 5.12 | Letting other people talk and talk without interrupting them. |  |  |  |  |  |
| 5.12 | Letting other people talk and talk without interrupting them. |  |  |  |  |  |
| 5.13 | Allowing others to speak too fast for everyone to understand them. |  |  |  |  |  |
| 5.14 | Not asking for more explanations when understanding becomes too difficult. |  |  |  |  |  |
| 5.15 | Other: |  |  |  |  |  |

5a. If you selected the item 15 'Other', please include your observations here.

## These questions are about your confidence in considering your and others' behaviours during group work. Please tick your answer for each question.

|  | Confidence of working in groups | Not confident at all | Not that confident | Reasonably confident | Extremely confident |
| --- | --- | --- | --- | --- | --- |
| 6.1 | How confident are you to engage in group discussion? |  |  |  |  |
| 6.2 | How confident are you to draw others into group discussion? |  |  |  |  |
| 6.3 | How confident are you to address the behaviour of someone who is dominating the discussion during group work? |  |  |  |  |
| 6.4 | How confident are you in moderating your own behaviour to benefit group discussion? |  |  |  |  |

## To what extent do you think group behaviours can influence your learning? Please tick your answer for each question.

|  |  | Strongly disagree | Somewhat disagree | Neither agree nor disagree | Somewhat agree | Strongly agree |
| --- | --- | --- | --- | --- | --- | --- |
| 7.1 | Group discussion with other students usually leads to a better understanding about a topic |  |  |  |  |  |
| 7.2 | The quality of the discussion is determined by the way the group members interact |  |  |  |  |  |
| 7.3 | The quality of the discussion is determined by knowledge of the group members |  |  |  |  |  |

1. Please add any additional thoughts about group work.

Thank you very much for your participation.
